# Enantioselective Dynamic Process Reduction of α- and β-Tetralone and Stereoinversion of Resulting Alcohols in a Selected Strain Culture

**DOI:** 10.1007/s00284-012-0143-2

**Published:** 2012-05-22

**Authors:** Tomasz Janeczko, Anna Panek, Alina Świzdor, Jadwiga Dmochowska-Gładysz, Edyta Kostrzewa-Susłow

**Affiliations:** 1 Department of Chemistry, Wrocław University of Environmental and Life Sciences, Norwida 25, 50-375 Wrocław, Poland; 2 Department of Cosmetology, Wrocław College of Physiotherapy, Kościuszki 4, 50-038 Wrocław, Poland

## Abstract

α-Tetralone and β-tetralone were subjected to biotransformation by 14 fungal strains. Enantiomeric purity of the products depended on the reaction time. 3-Day transformation of α-tetralone in *Absidia cylindrospora* culture gave *S*-(+)-1,2,3,4-tetrahydro-1-naftol of 92 % ee, whereas longer biotransformation time resulted in decrease of ee value. 3-Day transformation of β-tetralone by the same strain gave predominantly *S*-(−)-1,2,3,4-tetrahydro-2-naftol, whereas after 9 days of the reaction, the *R*-enantiomer with 85 % ee was isolated. Transformation of β-tetralone by *Chaetomium* sp*.* KCh 6651 gave pure (*S*)-(−)-1,2,3,4-tetrahydro-2-naftol in high yield at the concentration of 1 g/l. In this process, a non-selective carbonyl reduction was observed, followed by a selective oxidation of the *R*-alcohol.

## Introduction

Enantiomerically pure alcohols are useful chiral building blocks for asymmetric synthesis of bioactive compounds of complicated structures [9, 12, 22, 24]. For this reason, studies on effective way of their production on a preparative scale are undertaken. An asymmetric reduction of ketones using biocatalysts is a method that fulfills green chemistry requirements [6, 14, 20]. Employment of intact microorganisms’ cells, of both growing and resting-state cultures, eliminates problems with regeneration of co-factors cooperating with enzymes. Low costs and simplicity of the experimental procedure make microbial reduction of carbonyl group an interesting alternative to methods which use synthetic catalysts [4].

In order to find new biocatalysts that are capable to reduce enantiospecifically the aliphatic–aromatic ketones, we checked several strains of filamentous fungi and yeasts. We have chosen α- (**1**) and β-tetralone (**3**) as substrates for the tests because products of their reduction are used as chiral synthons in pharmaceutical industry [7, 23, 26]. (*S*)-α-Tetralol ((*S*)-**2**) is a substrate in synthesis of antidepressant medicines [21, 23], whereas enantiomerically pure (*S*)-β-tetralol ((*S*)-**4**) and its hydroxy and alkoxy derivatives may be employed in synthesis of 2-aminotetralins, the compounds which express activity toward dopamine, serotonin, and melatonin receptors [7, 26]. The substituted (*S*)-β-tetralol is a precursor of a drug used in cardiac arrhythmia [15, 18, 26]. Analogs of 2-aminotetralin have been used in the synthesis of drugs for the treatment of many central nervous system-related disorders [3] and in the synthesis of antifungal agents [25].

## Experimental

### Materials

The substrates: α-tetralone (**1**) and β-tetralone (**3**) were purchased from Sigma-Aldrich. All the microorganisms: *Coryneum betulinum* KCh 6534, *Absidia cylindrospora* KCh 336, *Saccharomyces brasiliensis* KCh 905, *Chaetomium* sp*.* KCh 6651, *Chaetomium* sp*.* KCh 6665, *Absidia coerulea* KCh 93, *Disculina betulina* KCh 6538, *Apignomia tiliae* KCh 6610, *Chalara* sp*.* KCh 6664, *Nectria galligena* KCh 6640, *Cryptosporiopsis radicola* KCh 6671, *Saccharomyces pastorianus* KCh 906, *Hanseniaspora uvarum* KCh 912, and *Endomyces fibulger* KCh 913 were isolated from forest environment (south Poland) from dead parts of leafy plants and identified in the Department of Forest Phytopathology of the University of Agriculture in Kraków, Poland. The strains were cultivated on a Sabouraud agar consisting of: aminobac 5 g, peptone K 5 g, glucose 40 g, and agar 15 g in 1 l of distilled water at 28 °C and pH 5.5, and stored in refrigerator at 4 °C.

### Analytical Methods

The course of biotransformation was controlled by means of TLC. Analytical TLC was carried out on silica gel G 60 F_254_ plates (Merck). Chromatograms were developed using hexane/acetone mixture (3:1 v/v) as the eluent. Compounds were detected by spraying the plates with 1 % Ce(SO_4_)_2_ and 2 % H_3_[P(Mo_3_O_10_)_4_] in 10 % H_2_SO_4_. Products were separated by column chromatography using silica gel (SiO_2_, Kieselgel 60, 230–400 mesh, 40–63 μm, Merck) and hexane/acetone mixture (3:1, v/v) as the developing system. Composition of biotransformation mixtures was established by GC. GC analysis was performed using a Hewlett-Packard 5890A (Series II) GC instrument fitted with a flame ionization detector. The chiral, capillary column (for **1**: Chirasil-Dex CB 25 m × 0.25 mm × 0.25 μm; temperature program 120 °C/1 min, gradient 1 °C min^−1^ to 145 °C, gradient 20 °C min^−1^ to 200 °C/5 min and for **3**: Chirasil-L-Val 25 m × 0.25 mm × 0.12 μm; temperature program 80 °C/1 min, gradient 1 °C min^−1^ to 110 °C, gradient 20 °C min^−1^ to 200 °C/5 min) was used to determinate the composition and enantiomeric excesses of product mixtures. Authentic reference samples of the racemic alcohol were prepared by reducing the ketones with sodium borohydride in methanol. The NMR spectra were recorded on a DRX 500 MHz Bruker spectrometer and measured in CDCl_3_. Optical rotations were measured on an Autopol IV automatic polarimeter (Rudolph). The absolute configuration of the products was determined by comparison of their optical rotational values with literature data.

### Screening Procedure

Erlenmeyer flasks (300 ml), each containing 100 ml of the medium consisting of 3 g glucose and 1 g aminobac dissolved in water, were inoculated with a suspension of microorganisms and then incubated for 3–7 days at 25 °C on a rotary shaker (190 rpm). After full growth of the culture (about 12 g of cell dry weight/l), 20, 40, 60, 80, and 100 mg of a substrate dissolved in 1 ml of acetone was added. After 1, 3, 6, and 9 days of incubation under the above conditions, portions of 10 ml of the transformation mixture were taken out and extracted with CHCl_3_ (3 × 10 ml). The extracts were dried over MgSO_4_, concentrated in vacuo, and analyzed by GC. All the experiments were repeated three times.

### Preparative Biotransformation

The same transformations were performed on the preparative scale in 2,000-ml flasks, each containing 500 ml of the cultivation medium. The cultures were incubated under the same conditions and then 200 mg of substrate dissolved in 2 ml of acetone was added to the grown *C. betulinum* culture (the strain selected for detailed studies). After 6 days of incubation, the mixtures were extracted with CHCl_3_ (3 × 300 ml), dried (MgSO_4_), and concentrated in vacuo. The transformation products were separated by column chromatography and analyzed (TLC, GC, ^1^H NMR).

### Spectral Data of Isolated Metabolites

It was proved that the obtained products were the secondary alcohols. The locations and orientations of the introduced hydroxyl groups were determined on the basis of the chemical shifts and the shapes of C*H*OH signals in the ^1^H NMR spectra.

#### (*S*)-1-tetralol (**2**)

Colorless oil [*α*]_*D*_^20 ^= +19.4° (*c* 2.5, CHCl_3_) (86 % ee), (lit. [*α*]_*D*_^25 ^= +23°, 96 % ee [2]); ^1^H NMR (CDCl_3_) δ (ppm): 1.76–2.01 (m, 4H, C*H*_2_ at C-2 and C-3); 2.73–2.82 (m, 2H, C*H*_2_ at C-4); 4.75 (t, 1H, *J* = 4.9 Hz, H-1); 7.03–7.37 (m, 3H, H-5, H-7 and H-8); 7.45 (1H, m, *W*_h_ = 12.5 Hz, H-6); GC: *R*_t_ (*S*) 23.0 min, *R*_t_ (*R*) 23.5 min.

#### (*S*)-2-tetralol (**4**)

Pale yellow oil [*α*]_*D*_^23^ = −59.2° (*c* 2.4, CHCl_3_) (100 % ee), (lit. [*α*]_*D*_^25^ = −20.8° (*c* = 0.25, MeOH, 29 % ee (*S*) [13]); ^1^H NMR (CDCl_3_) δ (ppm): 1.82 (dtd, 1H, *J* = 12.7, 9.1, 5.9 Hz, H-3_eq_); 2.05 (m, 1H, *W*_h_ = 27.4 Hz, H-3_ax_); 2.76 (dd, 1H, *J* = 16.2, 7.5 Hz, H-1_ax_); 2.86 (m, 1H, *W*_h_ = 15.2 Hz, H-4_ax_); 2.96 (dt, 1H, *J* = 17.0, 5.8 Hz, H-4_eq_); 3.09 (dd, 1H, *J* = 16.2, 4.9 Hz, H-1_eq_); 4.16 (m, 1H, *W*_h_ = 24.7 Hz, H-2); 7.10 (m, 4H, *W*_h_ = 25.6 Hz, H–Ar); GC: *R*_t_ (*S*) 26.9 min, *R*_t_ (*R*) 27.3 min.

## Results and Discussion

In our previous studies on enantiospecific reduction of α-tetralone (**1**) and β-tetralone (**3**), the biotransformations in *Fusarium culmorum* [16] and *Didymosphaeria igniaria* KCh 6670 [17] cultures were presented. The obtained results prompted us to test another group of available microorganisms.

Out of 14 fungal strains that were checked for bioreduction of α-tetralone (**1**) and β-tetralone (**3**), only a few (Tables 1, 2) expressed the desired activity.

**Table 1:** Microbial reduction of α-tetralone (**1**)

Microorganism	Substrate concentration (g/dm^3^)	Reaction time (days)	Substrate conversion (%)	Product (alcohol)	
				ee (%)	Absolute configuration
*Chaetomium* sp*.* KCh 6651	0.2	1	7	53	*S*
		3	13	90	*S*
		6	8	96	*S*
		9	2	98	*S*
*C. betulinum* KCh 6534	0.4	1	45	83	*S*
		3	63	84	*S*
		6	75	86	*S*
		9	81	88	*S*
*A. cylindrospora* KCh 336	0.2	1	42	91	*S*
		3	72	92	*S*
		6	56	82	*S*
		9	38	79	*S*
*A. coerulea* KCh 93	0.2	1	19	100	*S*
		3	48	74	*S*
		6	72	63	*S*
		9	76	50	*S*
*S. brasiliensis* KCh 905	0.2	1	89	4	*S*
		3	93	3	*S*
		6	96	1	*S*
		9	95	0	*S*
*Chaetomium* sp*.* KCh 6665	0.2	1	2	71	*S*
		3	6	71	*S*
		6	6	70	*S*
		9	7	70	*S*

**Table 2:** Microbial reduction of β-tetralone (**3**)

Microorganism	Substrate concentration (g/dm^3^)	Reaction time (days)	Substrate conversion (%)	Product (alcohol)	
				ee (%)	Absolute configuration
*Chaetomium* sp*.* KCh 6651	1	1	84	38	*S*
		3	96	63	*S*
		6	98	100	*S*
		9	100	100	*S*
*C. betulinum* KCh 6534	0.8	1	88	69	*S*
		3	98	85	*S*
		6	100	100	*S*
		9	100	100	*S*
*A. cylindrospora* KCh 336	0.6	1	42	41	*S*
		3	90	22	*S*
		6	98	31	*R*
		9	99	85	*R*
*S. brasiliensis* KCh 905	1	1	84	6	*S*
		3	95	7	*S*
		6	98	7	*S*
		9	100	7	*S*
*Chaetomium* sp*.* KCh 6665	0.2	1	4	85	*S*
		3	8	93	*S*
		6	9	98	*S*
		9	10	100	*S*

Neither α-tetralone (**1**) nor β-tetralone (**3**) were reduced in the following cultures: *D. betulina* KCh 6538, *A. tiliae* KCh 6610, *Chalara* sp*.* KCh 6664, *N. galligena* KCh 6640, *C. radicola* KCh 6671, *S. pastorianus* KCh 906, *H. uvarum* KCh 912, and *E. fibulger* KCh 913. The strain *A. coerulea* KCh 93 transformed exclusively α-tetralone.

Comparing the results presented in Tables 1 and 2, one can notice that β-tetralone (**3**) was transformed much quicker than α-tetralone (**1**) by the majority of the tested fungal strains. Moreover, much higher concentrations of the substrate **3** were tolerated compared to α-tetralone (**1**), and alcohol **4** was obtained with higher optical purity than product **2**. These results are different from the data reported in the literature, concerning reduction with the help of chiral catalysts, where **1** was more effectively reduced than **3** [5, 8].

Transformations of both substrates resulted in reduction of their carbonyl groups, leading mainly to the respective (*S*)-alcohols according to the Prelog rule.

In the culture of *C. betulinum* KCh 6534, we obtained (*S*)-(+)-1,2,3,4-tetrahydro-1-naftol ((*S*)-**2**) in 75 % yield and the enantiomeric excess above 86 %. This result was achieved after 6 days of the biotransformation. In the culture of *A. cylindrospora* KCh 336, the same product (*S*)-**2** was obtained after 3 days with much higher optical purity—above 92 % at 72 % substrate conversion.

In the culture of *A. coerulea* KCh 93, the yield of reduction of **1** increased with time, but the enantiomeric purity of formed alcohol (*S*)-**2** decreased. After 1 day of transformation, the product was obtained with the ee as high as 100 %, but in low yield (19 %). After 9 days of transformation, (*S*)-**2** was obtained in 76 % yield, but in low ee (50 %).

Influence of the reaction time on enantiomeric purity of the product was also observed in the case of transformation of β-tetralone (**3**) by the strain of *A. cylindrospora* KCh 336 (Table 2). After 3 days of the transformation, the *S*-enantiomer of **4** prevailed in the reaction mixture, whereas after 9-day transformation its isomer ((*R*)-**4**) was isolated with 85 % of enantiomeric purity (the (*R*)-**4** alcohol represents the anti**-**Prelog product).

**Fig. 1:**
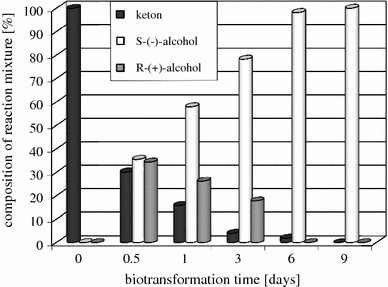
Time dependence of the transformation of β-tetralone (**3**) in *Chaetomium* sp*.* KCh 6651 culture

Another interesting result was observed in the transformation of β-tetralone (**3**) by *Chaetomium* sp. KCh 6651. In this process, at substrate concentration of 1 g/l, after 6 days we obtained enantiomerically pure (*S*)-(−)-1,2,3,4-tetrahydro-2-naftol ((*S*)-**4**) at 98 % ketone conversion. Tracing the progress of the reaction with time (Fig. 1), we observed that the used strain expresses reductases of different enantioselectivities (one showing Prelog preference and the other showing “anti-Prelog” preference). After 12 h of the reduction, the enantioselectivity of this reaction was extremely low. In the reaction medium, both alcohols were present giving practically racemic mixture. Next, when the incubation was continued, the content of *R*-enantiomer ((*R*)-**4**) gradually decreased. These facts indicate that (*R*)-**4** was oxidized to the ketone **3**, but (*S*)-**4** could not be oxidized under the reaction conditions.

**Scheme 1:**
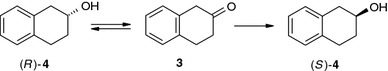
Transformation of β-tetralone (**3**) in the culture of *Chaetomium* sp*.* KCh 6651

Monitoring of this biotransformation in time (by GC analysis) confirmed the conclusion that at first non-selective carbonyl group reduction occurs, followed by selective oxidation of the *R*-alcohol to β-tetralone (**3**). Thus, one possible mechanism of stereoinversion is that oxidoreduction between (*R*)-**4** and **3** is reversible and the reduction of **3** is irreversible (Scheme 1).

The corresponding time profiles for the transformation of α-tetralone in the culture of this strain do not feature such prominent variations. In this case, after the third day of transformation, the substrate conversion of 13 % was observed. However, after even longer elapsed time, there was decrease in the percentage of the alcohol in the biotransformation mixture, which indicates increase in the oxidation process rate relative to the reduction.

Comparison of the conversion rates of both substrates in the *Chaetomium* sp*.* KCh 6651 strain culture leads to the conclusion that the substrate **3** undergoes reduction more efficiently. The alcohol **4** undergoes oxidation with slightly higher enantiospecificity with respect to the alcohol **2**.

Transformation of **3** in the culture of *C. betulinum* KCh 6534 gave optically pure (*S*)-(−)-1,2,3,4-tetrahydro-2-naftol ((*S*)-**4**) in 100 % yield after 6-day biotransformation process. In the case of this strain, as in the culture of *Chaetomium* sp*.* KCh 6651 described above, lower enantiomeric excesses of the formed alcohol (*S*)-**4** were initially (after 1 and 3 days) observed. The substrate **1** underwent reduction process in the *C. betulinum* KCh 6534 strain culture slower with respect to the compound **3**. In this case, however, so much pronounced stereoinversion was not observed—the enantiomeric excess increased from 83 % after the first transformation day to 88 % in the ninth day.

In summary, the manuscript presents the biotransformation of α-tetralone (**1**) and β-tetralone (**3**) with particular emphasis on time variation of reaction mixture composition—a feature closely related to enantiospecificity of oxidation of alcohols formed previously in either enantioselective or non-enantioselective reduction (*Chaetomium* sp. KCh 6651, substrate **3**).

There are numerous experiments described in the literature related to enantioselective oxidation of one enantiomer using racemic mixture of phenylethanols as the substrate [1, 10, 11, 19]. Only a few manuscripts have so far reported concurrent percentage variations of two enantiomers of alcohols formed during reduction of pro-chiral ketones [16, 17].

In our experiments, we noted the sustained selectivity of dehydrogenases both in reduction and oxidation processes. For example, during transformation of β-tetralone in the culture of *A. cylindrospora*, the ketone is reduced to an *S*-alcohol with high enantiomeric excess, and simultaneously the obtained *S*-alcohol is at once oxidized back to the ketone with even higher enatioselectivity. Such mechanism of transformation leads to the observed decrease of enantiomeric excess of the initially formed *S*-alcohol after sufficiently long time of biotransformation process.
